# Predictors of poorly developed coronary collateral circulation in patients with subclinical hypothyroidism suffered from chronic stable angina

**DOI:** 10.21542/gcsp.2019.10

**Published:** 2019-09-20

**Authors:** Mohamed Khalfallah, Enas Draz, Khaled Shalaby, Yasser Mostafa Hafez

**Affiliations:** 1 Department of Cardiovascular Medicine, Faculty of Medicine, Tanta University, Tanta, Egypt; 2 Department of Internal Medicine, Faculty of Medicine, Tanta University, Tanta, Egypt

## Abstract

**Background.** The development of coronary collaterals is variable among patients with coronary artery disease and remains incompletely understood. We aimed to demonstrate the predictors of poorly developed coronary collateral circulation (CCC) in patients with subclinical hypothyroidism suffered from chronic stable angina.

**Methods.** The study was conducted on 226 patients with subclinical hypothyroidism suffered from chronic stable angina, coronary angiography documented total occlusion at any major coronary artery or coronary artery lumen diameter stenosis >90%. Patients were divided into two groups according to grade of CCC, group A: 138 patients with (good collaterals) and group B: 88 patients with (poor collaterals). To classify CCC, we used Rentrop’s classification.

**Results.** Multivariate regression analysis was performed and identified the independent predictors of poor coronary collaterals: N/L ratio (OR 0.413, CI 95% [0.172–0.993], p = 0.048), and TSH (OR 2.511, CI 95% [1.784–3.534], p = 0.001). The ROC analysis provided a cut-off value of >4.6 for N/L ratio, and >9 µIU/mL for TSH to predict poor coronary collaterals.

**Conclusion.** An elevated level of N/L ratio >4.6 and TSH level >9 µIU/mL were the independent predictors of poorly developed CCC in patients with subclinical hypothyroidism suffered from chronic stable angina.

## Introduction

Coronary artery disease (CAD) is one of the most common causes of morbidity and mortality worldwide. The development of coronary collateral circulation (CCC) is considered a physiological adaption of the myocardium to circumvent ischemia, also serves as a natural bypass for blood supplying myocardium jeopardized by ischemia^1,2^. The number of collaterals and the extent of their coverage are associated with improved survival in patients with CAD. Although these anastomoses are often incapable of restoring flow to normal levels, but help in alleviating episodes of myocardial ischemia, enhancing residual myocardial contractility, reducing infarct size, preserving left ventricular function, reducing coronary atherosclerotic disease progression and decreasing mortality^3,4^. In normal individuals, coronary collaterals present between the large coronary arteries, but it cannot be visualized on coronary angiography because there is no significant volume of blood inside^5,6^.

The development of CCC involves the development of capillaries (angiogenesis) and muscular collateral arteries (arteriogenesis). Arteriogenesis is responsible for the development of epicardial CCC^7^. The mechanisms for different individual ability to develop CCC are still unclear. It is well known and accepted that their active functioning is directly related to the occurrence of severe and recurrent ischemia, but other factors may also influence their development, e.g., the presence of diabetes mellitus, hypertension, endothelial dysfunction and levels of inflammatory cells were all suggested as potential determinants of collateral development. The inflammatory process plays an important role at all stages of CAD^8^. Impaired development of CCC may be associated with increase in acute phase reactants, e.g., C-reactive protein (CRP), and indicates the role of inflammation in this process^9^. It is also well known that leukocyte activation occurs during an inflammatory reaction. White blood cell subtypes, especially the neutrophil-to-lymphocyte ratio (N/L ratio), can be used as an indicator of systemic inflammation and may be associated with poor CCC development.

Subclinical hypothyroidism is a common disease that is characterized by high level of thyroid-stimulating hormone (TSH) with free thyroxin concentrations within the lower normal reference range^10^. Vascular smooth muscle cells are an important target of thyroid hormones, and high levels of TSH may lead to an increase in the risk of adverse cardiovascular events, such as micro vascular endothelial dysfunction, inhibition of vascular smooth muscle cell migration^11,12^, acceleration of atherosclerosis, and an increase in the risk of CAD^13^. Endothelial dysfunction is characterized by an imbalance between vasodilator and vasoconstrictor activity and may affect microvascular or epicardial vessels^14^.

The underlying physiological and pathological factors influencing the development of CCC remain unclear. In the present study, our objective was to investigate the most important predictors of poor CCC, as evaluated by coronary angiography in patients with subclinical hypothyroidism suffered from chronic stable angina. We must gain further insight into these factors as CCC is considered one of the compensatory mechanisms for amelioration of ischemia, particularly in patients for whom revascularization is not amenable.

### Patients and methods

The present study was conducted on 226 patients with subclinical hypothyroidism suffered from chronic stable angina. Coronary angiography documented total occlusion at any major coronary artery or coronary artery lumen diameter stenosis >90%. All patients were referred to our cardiovascular department for elective coronary angiography during the period January 2016 to August 2018. Informed consent was obtained from all participants in this research. Every patient included in the study had a code number pointed to his name, address and telephone number and the data was saved in a special file. The study was approved by the local ethical committee, and was in accordance with the principles of the declaration of Helsinki II.

Patients were divided into two groups according to grade of CCC. Group A: (good collaterals) and Group B: (poor collaterals). In the present study we excluded patients with history of myocardial infarction, patients with severe hepatic or renal impairment (serum creatinine level more than 1.5 mg/dL), patients with severe anemia, patients with decompensated heart failure, patients with concomitant inflammatory and neoplastic diseases, patients with coronary artery bypass grafting, patients with clinical thyroid disorders such as overt hyper or hypothyroidism and thyroiditis and patients who used the following medications (amiodarone, lithium, propylthiouracil, corticosteroids and immunosuppressive drugs).

Baseline clinical, demographic data, medical histories and detailed risk profile were recorded from all patients. Full physical examination was performed on all patients. Blood pressure was measured with a standard mercury sphygmomanometer with an appropriately-sized cuff. Resting standard twelve lead ECG was done for all patients. Transthoracic echocardiography was performed in accordance with the recommendations of the American Society of Echocardiography. Left ventricular ejection fraction (LVEF) was calculated from linear dimensions measurements by M-mode, linear internal dimensions of left ventricle were measured from the parasternal long axis view carefully obtained perpendicular to the left ventricle long axis and measured at the level of mitral valve leaflet tips.^15^

### Laboratory measurement

Before coronary angiography, all individuals were subjected to a 12 hour overnight fast, followed by collection of venous blood samples. Laboratory investigations included: complete blood count, hematological parameters such as hemoglobin, platelets, white blood cells and their subtypes. N/L ratio was measured by dividing total number of neutrophils by lymphocytes number. Lipid profile, blood urea, serum creatinine and serum uric acid were also measured. Serum thyroid hormones including free triiodothyronine (FT3), free thyroxine (FT4) and TSH levels were evaluated by electro chemiluminescence immunoassay method (ECLIA) using E2010 (elecsys module) immunoassay analyzers (Cobas e 411 analyzer). Subclinical hypothyroidism represents a state with increased values of TSH and normal values of FT3 (2.3–4.2 pg/mL) and FT4 (0.8–1.8 ng/dL). The diagnosis was made based on the results of laboratory findings when the level of TSH reaches values above 4.0 µIU/mL.^16^

### Standard coronary angiography and coronary collaterals scoring

Coronary angiography had been performed using femoral or radial approach. Coronary angiograms and also collateral grading were examined by interventional cardiologists who were blinded to the clinical characteristics, laboratory results of the patients and study design. Coronary collaterals were graded according to the Cohen-Rentrop method: grade 0, no filling of any collateral vessels; grade 1, filling of side branches of the artery to be perfused by collateral vessels without visualization of epicardial segment; grade 2, partial filling of the epicardial artery by collateral vessels; and grade 3, complete filling of the epicardial artery by a collateral vessel. Patients with grade 0 to 1 were classified as poor coronary collaterals and patients with grade 2 to 3 were classified as good coronary collaterals.^17^

### Statistical analysis

Statistical analysis was performed using SPSS 20 (IBM, Armonk, NY, United States of America). Quantitative data were expressed as mean ± standard deviation (SD). Qualitative data were expressed as frequency and percentage. Student’s t-test was used to test significance between two groups in quantitative data. Chi-square test of significance was used in order to compare proportions between two qualitative parameters. P value <0.05 was considered statistically significant. Multivariate regression analysis was done to detect the independent predictors for poor coronary collateralization. Receiver Operating Characteristic curve (ROC-curve) analysis was performed for detection of sensitivity, specificity, positive and negative predictive values.

## Results

The study included 226 subclinical hypothyroidism patients with stable coronary artery disease who presented for elective coronary angiography and documented total occlusion at any major coronary artery or coronary artery lumen diameter stenosis >90%. Patients were divided into two groups according to grade of CCC. Group A were patients with grade 3 or grade 2 coronary collaterals were defined as the group with good collaterals which included 138 patients with a mean age of 57.10 ± SD 9.02 years, 84 males (60.9%). Group B patients had grade 1 or grade 0 coronary collaterals and were defined as the group with poor collaterals. They included 88 patients with a mean age of 55.69 ± SD 9.14 years, 47 males (53.4%). Demographic, clinical characteristics, hematological, biochemical parameters and angiographic results of all patients are summarized in Table 1. There was no statistically significant difference between the groups regarding age, sex, hypertension, diabetes mellitus, smoking, dyslipidemia and the previous medications of the patients except previous statin use. Patients who were on high intensity statin therapy showed better coronary collateral grade than patients who were on low or moderate intensity statin therapy (p = 0.009).

With respect to laboratory results, there was also no statistically significant difference between the groups regarding hemoglobin level, platelet count, random blood sugar, blood urea, serum creatinine, lipid profile, FT3 and FT4. However, there was statistically significant difference between the groups with higher N/L ratio in poor collaterals group compared to good collaterals group, (5.05 ± 0.71 vs. 3.91 ± 0.73, p value = 0.001). Also serum uric acid was higher in the poor collaterals group compared to the good collaterals group, (5.77 ± 1.51 vs. 5.38 ± 1.36 mg/dL, p value = 0.047).

**Table 1:** Demographic, clinical characteristics, laboratory results and angiographic results of all patients in both groups.

	Total (n = 226)	Good collaterals	Poor collaterals	p-value
Age, years	56.55 ± 9.08	57.10 ± 9.02	55.69 ± 9.14	0.256
Male gender, n (%)	131 (58.0%)	84 (60.9%)	47 (53.4%)	0.268
Hypertension, n (%)	133 (58.8%)	86 (62.3%)	47 (53.4%)	0.184
Diabetes mellitus, n (%)	108 (47.8%)	67 (48.6%)	41 (46.6%)	0.774
Smoking, n (%)	112 (49.6%)	66 (47.8%)	46 (52.3%)	0.514
Dyslipidemia, n (%)	194 (85.8%)	122 (88.4%)	72 (81.8%)	0.166
Heart rate (bpm)	63.93 ± 4.79	63.70 ± 4.25	64.28 ± 5.55	0.376
Systolic BP, mmHg	143.29 ± 24.13	144.06 ± 24.04	142.09 ± 24.35	0.551
Diastolic BP, mmHg	82.79 ± 9.42	83.48 ± 9.62	81.70 ± 9.06	0.168
LVEF, (%)	54.55 ± 7.93	56.67 ± 6.69	51.24 ± 8.61	0.001^*^
**Laboratory results**				
Hemoglobin, g/dL	14.27 ± 10.61	14.79 ± 13.54	13.47 ± 1.20	0.364
WBC count [×10^3^/µl]	7.26 ± 1.99	7.38 ± 2.06	7.07 ± 1.87	0.257
Platelets count [×10^3^/µl]	272.7 ± 86.9	271.2 ± 82.2	275.0 ± 94.3	0.751
Neutrophil [×10^3^/µl]	6.11 ± 0.95	5.59 ± 0.76	6.93 ± 0.57	0.001^*^
Lymphocyte [×10^3^/µl]	1.43 ± 0.16	1.45 ± 0.16	1.38 ± 0.13	0.001^*^
N/L ratio	4.35 ± 0.91	3.91 ± 0.73	5.05 ± 0.71	0.001*
Blood glucose, mg/dl	208.6 ± 104.2	205.0 ± 107.4	214.2 ± 99.2	0.522
Blood urea, mg/dl	22.04 ± 7.83	22.33 ± 8.26	21.59 ± 7.15	0.489
Serum creatinine, mg/dl	1.14 ± 0.17	1.14 ± 0.16	1.14 ± 0.18	0.997
Total cholesterol, mg/dl	276.39 ± 73.72	282.99 ± 72.04	266.04 ± 75.52	0.092
HDL, mg/dl	38.94 ± 8.44	39.28 ± 8.89	38.42 ± 7.70	0.458
LDL, mg/dl	132.37 ± 27.40	132.25 ± 30.89	132.57 ± 20.95	0.932
Triglycerides, mg/dl	181.71 ± 38.91	183.79 ± 39.01	178.45 ± 38.76	0.316
Serum uric acid, mg/dl	5.54 ± 1.43	5.38 ± 1.36	5.77 ± 1.51	0.047^*^
TSH, µIU/mL	9.75 ± 2.96	8.22 ± 2.06	12.15 ± 2.54	0.001^*^
FT3, pg/mL	3.13 ± 0.74	3.23 ± 0.74	2.97 ± 0.72	0.011^*^
FT4, ng/dL	1.54 ± 0.31	1.53 ± 0.32	1.55 ± 0.30	0.576
**Previous medications**				
Beta blockers, n (%)	205 (90.7%)	126 (91.3%)	79 (89.8%)	0.699
CCB, n (%)	56 (24.8%)	39 (28.3%)	17 (19.3%)	0.129
ACE I/ ARB, n (%)	188 (83.2%)	112 (81.2%)	76 (86.4%)	0.308
Antiplatelet, n (%)	187 (82.7%)	118 (85.5%)	69 (78.4%)	0.169
Statins, n (%)	188 (83.2%)	117 (84.8%)	71 (80.7%)	0.422
High intensity statins	110 (48.7%)	77 (65.8%)	33 (46.5%)	0.009^*^
Low or moderate intensity statins	78 (34.5%)	40 (34.2%)	38 (53.5%)	
Nitrates, n (%)	134 (59.3%)	77 (55.8%)	57 (64.8%)	0.180
**Angiographic results**				
Left main	4 (1.8%)	1 ( 0.7% )	3 (3.4%)	0.136
LAD	148 (65.5%)	85 (61.6%)	63 (71.6%)	0.123
LCX	57 (25.2%)	28 (20.3%)	29 (33%)	0.033^*^
RCA	77 (34.1%)	41 (29.7%)	36 (40.9%)	0.083
**Number of vessels**				
1	179 (79.2%)	124 (89.9%)	55 (62.5%)	0.001^*^
2	34 (15%)	12 (8.7%)	22 (25%)	
3	13 (5.8%)	2 (1.4%)	11 (12.5%)	
**Grade of collaterals**				
0	37 (16.4%)	0 (0%)	37 (42%)	0.001^*^
1	51 (22.6%)	0 (0%)	51 (58%)	
2	81 (35.8%)	81 (58.7%)	0 (0%)	
3	57 (25.2%)	57 (41.3%)	0 (0%)	

**Notes.**

BP blood pressure LVEF left ventricular ejection fraction WBC white blood cell N/L ratio neutrophil/lymphocyte ratio HDL high density lipoprotein LDL low-density lipoprotein TSH thyroid stimulating hormone FT3 free triiodothyronine FT4 free thyroxine CCB calcium channel blockers ACE I/ARB angiotensin converting enzyme inhibitors/angiotensin receptor blockers LAD left anterior descending LCX left circumflex RCA right coronary artery

* significant p-value.

For serum TSH levels there was statistically significant difference between both groups, with higher TSH levels in the poor collaterals group (12.15 ± 2.54 vs. 8.22 ± 2.06 µIU/mL, p value = 0.001). Among 138 patients with good CCC, 57 patients had Rentrop grade 3 and 81 patients had Rentrop grade 2. Among 88 patients with poor CCC, 51 patients had Rentrop grade 1 and 37 patients had had Rentrop grade 0.

**Figure 1.**
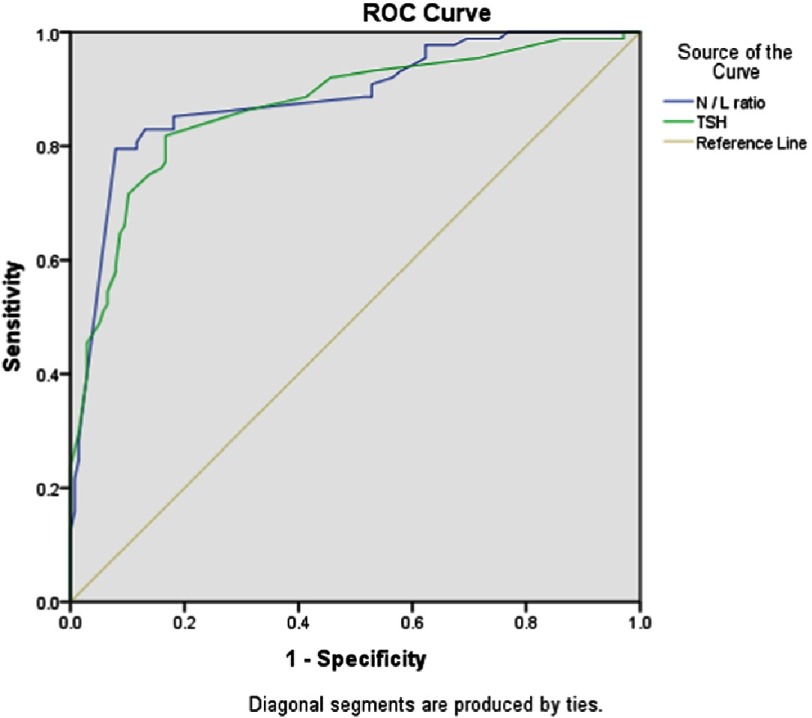
ROC curve analysis of the independent predictors of poor coronary collateral.

Multivariate regression analysis was performed and identified the independent predictors of poor coronary collaterals: N/L ratio OR 0.413 (CI 95% 0.172–0.993, p = 0.048), and TSH OR 2.511 (CI 95% [1.784–3.534], p = 0.001). ROC curve analysis was performed to detect cut-off value, sensitivity and specificity as shown in Tables 2 and 3, and Figure 1. The ROC analysis provided a cut-off value of >4.6 for N/L ratio to predict poor coronary collaterals with sensitivity 82.9% and specificity 86.9%. As regarding to thyroid hormones ROC analysis provided a cut-off value of >9 µIU/mL for TSH to predict poor coronary collaterals with sensitivity 81.8% and specificity 83.3%.

**Table 2:** Multivariate regression analysis of the independent predictors of poor coronary collaterals.

	Multivariate regression analysis	
	Adjusted OR (95% CI)	p-value
Neutrophil count	0.574 (0.243–4.521)	0.207
Lymphocytic count	1.025 (0.417–5.627)	0.085
N/L ratio	0.413 (0.172–0.993)	0.048^*^
Serum uric acid	0.547 (0.143–5.210)	0.241
TSH	2.511 (1.784–3.534)	0.001^*^
FT3	1.203 (0.042–5.320)	0.109
LVEF	1.743 (0.329–2.521)	0.141

**Notes.**

N/L ratio neutrophil/lymphocyte ratio TSH thyroid stimulating hormone FT3 free triiodothyronine LVEF left ventricular ejection fraction

* significant p-value.

**Table 3:** ROC curve analysis of the independent predictors of poor coronary collaterals.

	Cut-off value	Sensitivity	Specificity	PPV	NPV
N/L ratio	>4.6	82.9%	86.9%	80.2%	88.9%
TSH, µIU/mL	>9	81.8%	83.3%	75.8%	87.8%

**Notes.**

PPV positive predictive value NPV negative predictive value N/L ratio neutrophil/lymphocyte ratio TSH thyroid stimulating hormone

## Discussion

Coronary collateral circulation plays an important role in obstructive CAD as an adaptive response to myocardial ischemia. In the human heart, the major epicedial coronary arteries communicate with one another by means of anastomotic channels. Normally, these small collateral arteries allow very little blood flow. However, if arterial obstruction induces a pressure gradient across such a channel, then with time, the collateral vessel may dilate and serve as an alternative blood conveying circuit to the ischemic myocardium.

Endothelium activated by shear stress produces and releases adhesion molecules, accumulated monocytes and macrophages produce a variety of angiogenic growth factors and proteases. These bioactive substances contribute to coronary collateral growth^18^. Rentrop et al.^19^ demonstrated that the prevalence of angiographically demonstrable CCC increased as the lesion of collateral receiving coronary artery increased beyond 70% diameter, narrowing during elective coronary angioplasty.

There are many studies demonstrating the importance of coronary collaterals and their protective effect. Cohen and Rentrop^20^ found that coronary collaterals may help to protect the myocardium in patients with CAD by limiting myocardial ischemia during coronary occlusion. Fukai et al.^21^ found that well-developed coronary collaterals may minimize the infarct area and predict the presence of viable myocardium in patients with a history of myocardial infarction. Sabia et al.^22^ demonstrated that the myocardium may remain viable for a prolonged period in patients with a recent myocardial infarction and an occluded infarct related coronary artery in the presence of collaterals. Billinger et al.^23^ showed that patients with poor CCC experience higher incidence of acute coronary syndrome than those with good collaterals.

The inflammatory process plays an important role at all stages of coronary atherosclerosis from initiation through progression and also in the thrombotic complications of this disease. The N/L ratio is a combination of two independent markers of inflammation, the ratio between the number of neutrophils and the number of lymphocytes provides a simple method for assessment of the inflammatory status and prognosis in patients with CAD. In our study, there was a significant relationship between elevated levels of N/L ratio and poor coronary collaterals, The ROC analysis provided a cut-off value of >4.6 for N/L ratio to predict poor coronary collaterals with sensitivity 82.9% and specificity 86.9%.

In agreement with our results, Akın et al.,^24^ investigated predictors of poor coronary collaterals in a cohort of 248 patients who had high-grade coronary stenosis or occlusion. They used the Rentrop classification to classify CCC. They reported that patients with poorly developed CCC had significantly higher N/L ratio compared with those with well- developed CCC, (4.2 ± 2.8 vs. 3 ± 3.1, p=0.001). They concluded that an elevated level of N/L ratio is independently associated with a significant impairment in coronary collateralization.

High levels of TSH may lead to microvascular endothelial dysfunction, inhibition of vascular smooth muscle cell migration^11,12^, acceleration of atherosclerosis, and an increased risk of CAD. In the present study we noticed a significant relationship between high levels of serum TSH and poor coronary collaterals. The ROC analysis provided a cut-off value of >9 µIU/mL for TSH to predict poor coronary collaterals with sensitivity 81.8% and specificity 83.3%. Our results were similar to the results of Balli et al.,^25^ who studied the relationship between serum thyroid hormones levels and coronary collateral circulation in patients with stable CAD and found that there was statistically significant difference between good collaterals group and poor collaterals group as regard to the level of serum TSH (P value = 0.001). Yoneda et al.,^12^ showed that injecting TSH in conduit arteries resulted in significant impairment of endothelial vasodilatation and peripheral vascular resistance increase, which may contribute to poor coronary collaterals development.

The grade of coronary collaterals was directly related to the dose of statin therapy. Patients who were on high-intensity statin therapy showed better coronary collateral grade than patients who were on low- or moderate-intensity statin therapy with (p = 0.009). In agreement with our results Dincer et al, who studied the association between the dosage and duration of statin treatment with coronary collateral development among 400 patients, reported that patients who were on statin therapy for more than 3 months had significantly better collateral development (p = 0.002). Statin therapy had no effect on collateral development in patients having <10 mg atorvastatin-equivalent dose (p = 0.13)^26^.

### Study limitations

This study has the following limitations. First, the number of the patients in this study was relatively small. Also this study is a single-center study, which does not reflect the whole population, so other multicenter studies will help to validate the results. Coronary collateralization progresses over many years and we can’t determine the precise time of fully collateral development. In this study, our patients did not undergo invasive hemodynamic measurements as the gold standard for measuring coronary collateralization is intravascular hemodynamic assessment (usually collateral flow index; either pressure or velocity-derived. Also, the Rentrop classification has its limitations, as small microvascular caliber vessels may not be visualized angiographically. Finally, we did not assess oxidative markers, cytokines, and pro-arteriogenic markers, as they might give more information on the role of endothelial dysfunction, angiogenesis and its relation to coronary collateral development.

## Conclusion

Presence of good coronary collaterals is important for patients with obstructive CAD, particularly in patients for whom coronary revascularization is unsuitable. Our study showed the most important independent predictors of poor coronary collateral development. An elevated level of N/L ratio >4.6 as a marker of inflammation is independently associated with a significant impairment in CCC. TSH level >9 µIU/mL should be considered as a modifiable risk factor for poor coronary collateral development in patients with subclinical hypothyroidism suffered from chronic stable angina. This study will lead to more evaluation and consideration of the effects of subclinical hypothyroidism on cardiac morbidity and coronary artery disease progression, which in turn will be reflected on guidelines for early screening of thyroid disorders in ischemic heart disease. Also it will open the door for further studies to evaluate the efficacy of therapy and the target TSH level that can decrease or prevent CAD progression.

## Conflict of interest

The authors declare no conflicts of interest.
